# Calcium Imaging of Parvalbumin Neurons in the Dorsal Root Ganglia

**DOI:** 10.1523/ENEURO.0349-18.2019

**Published:** 2019-08-01

**Authors:** Marie C. Walters, Martha J. Sonner, Jessica H. Myers, David R. Ladle

**Affiliations:** Department of Neuroscience, Cell Biology, and Physiology, Boonshoft School of Medicine, Wright State University, Dayton, OH 45435

**Keywords:** calcium imaging, DRG, parvalbumin, sensory, transgenic

## Abstract

We investigated the calcium dynamics of dorsal root ganglion (DRG) neurons using transgenic mice to target expression of the genetically encoded calcium indicator (GECI), GCaMP6s, to a subset of neurons containing parvalbumin (PV), a calcium-binding protein present in proprioceptors and low-threshold mechanoreceptors. This study provides the first analysis of GECI calcium transient parameters from large-diameter DRG neurons. Our approach generated calcium transients of consistent shape and time-course, with quantifiable characteristics. Four parameters of calcium transients were determined to vary independently from each other and thus are likely influenced by different calcium-regulating mechanisms: peak amplitude, rise time (RT), decay time, and recovery time. Pooled analysis of 188 neurons demonstrated unimodal distributions, providing evidence that PV+ DRG neurons regulate calcium similarly as a population despite their differences in size, electrical properties, and functional sensitivities. Calcium transients increased in size with elevated extracellular calcium, longer trains of action potentials, and higher stimulation frequencies. RT and decay time increased with the addition of the selective sarco/endoplasmic reticulum calcium ATPases (SERCA) blocker, thapsigargin (TG), while peak amplitude and recovery time remained the same. When elevating bath pH to 8.8 to block plasma-membrane calcium ATPases (PMCA), all measured parameters significantly increased. These results illustrate that GECI calcium transients provide sufficient resolution to detect changes in electrical activity and intracellular calcium concentration, as well as discern information about the activity of specific subclasses of calcium regulatory mechanisms.

## Significance Statement

This study bridges two research fields by applying calcium imaging and transgenic mouse approaches to address, for the first time, questions relevant to motor system scientists regarding proprioceptive neuron physiology. We characterize the average parameters of calcium transients from parvalbumin (PV)+ dorsal root ganglion (DRG) neurons at a population level, and describe the diversity in calcium dynamics between cells, animals, and sexes. In addition to providing a detailed analysis of PV+ neuron calcium homeostasis, the application of calcium imaging in this study expands the repertoire of tools available to electrophysiologists investigating proprioceptive function.

## Introduction

Intracellular calcium regulation is a core feature of neuronal function, as calcium modulates excitability, neurotransmission, and other essential cellular processes (Brini et al., 2014). The connection between calcium homeostasis and neuronal physiology is underscored in the dorsal root ganglion (DRG), where peripheral sensory neurons have been shown to regulate calcium differently depending on their size and functional classification (Fuchs et al., 2005; Lu et al., 2006). Although studies have delineated calcium dynamics in nociceptive neurons (Gemes et al., 2011, 2012; Scheff and Gold, 2015) and in large DRG neurons (Fuchs et al., 2005; Duncan et al., 2013), characterization of calcium dynamics in other functionally defined DRG subpopulations is lacking. Using a transgenic animal model, the present study more precisely examines the calcium dynamics of specific DRG subpopulations by isolating low-threshold afferents, primarily proprioceptors, from other DRG neurons.

Unlike other subclasses of DRG neurons that can be identified in calcium imaging experiments by their responses to capsaicin (Fuchs et al., 2005) or ability to bind IB4 (Scheff et al., 2013), living proprioceptors can only be distinguished from other DRG neurons by unique electrophysiological responses and gene expression patterns. Thus, it is difficult to study the calcium dynamics of proprioceptors using dissociated cultures and traditional synthetic dyes. Genetically encoded calcium indicators (GECIs) permit targeted expression of calcium sensors to specific cell types. Notably, the vast majority of proprioceptive afferents express the calcium-binding protein parvalbumin (PV; Zhang et al., 1990; de Nooij et al., 2013). Furthermore, ∼90% of PV-containing (PV+) DRG neurons are proprioceptors (de Nooij et al., 2013). This study investigates the calcium dynamics of this population by collecting optical recordings from DRGs of transgenic mice that express calcium indicators only in PV+ neurons.

Proprioceptive neurons provide feedback critical to coordinated movement and posture. Previously, proprioceptive function has been studied using electrophysiological approaches, yielding information about the electrical activity and firing properties of healthy proprioceptors (Vincent et al., 2017), as well as elucidating proprioceptive abnormalities after peripheral nerve injury (Bullinger et al., 2011). Calcium imaging with GECIs can expand our knowledge of proprioceptive function to encompass calcium dynamics. In addition, the present technique isolates intrinsic properties of proprioceptors from the confounding influences of muscle spindles, muscle fibers, and spinal cord circuitry that influence mechanosensory encoding. Thus, employing calcium imaging to study proprioceptive function represents a valuable addition to information collected via electrophysiology.

The latest generation of GECIs offer increased sensitivity over non-ratiometric synthetic dye indicators and permit targeted expression of calcium sensors to specific cell types (Chen et al., 2013). Although several recent studies have used GECIs in the DRG (Emery et al., 2016; Kim et al., 2016; Smith-Edwards et al., 2016; Chisholm et al., 2018; Jayaraj et al., 2018), it has yet to be determined whether GECI transients, with relatively slow response kinetics (Rose et al., 2014), can provide insights into the calcium dynamics of somatosensory neurons. Studies using calcium indicator dyes have quantified calcium transient parameters in greater detail, and generated information about the contributions of specific calcium regulatory mechanisms to various phases of the transient (Scheff et al., 2013; Guo et al., 2017). We hypothesized that GECIs can also be used to monitor calcium dynamics in PV+ DRG neurons in an *ex vivo* preparation. High-speed calcium imaging of individual neurons enabled quantification of several aspects of the GECI calcium transient, including peak amplitude, rise time (RT), and decay times. These characteristics were then examined to determine the degree to which GECI calcium transients vary between cells, animals, and sexes among a physiologically similar population of neurons. Finally, sarco/endoplasmic reticulum calcium ATPases (SERCA) and plasma-membrane calcium ATPases (PMCA) were blocked to determine whether GECI calcium transients could detect and resolve activity changes of specific calcium regulatory mechanisms.

This study details the average characteristics of action potential-induced calcium transients from PV+ DRG neurons. Data were also examined at a global level to determine whether the calcium transients of PV+ neurons were homogenous or whether there were subpopulations that regulate calcium differently. In addition to describing new functional properties of PV+ neurons, the approach used in this study makes it possible to examine proprioceptive function without stimulating the peripheral or central connections of proprioceptive neurons; a tool that is particularly beneficial in the case of peripheral nerve injury where peripheral and central connections are disrupted. Finally, the results from blocking SERCA or PMCA demonstrate that GECIs can be used to extract meaningful information about the activity of calcium-regulating mechanisms when a baseline or sham condition is used for comparison.

## Materials and Methods

### Animals

All animal experimental procedures were conducted under the approval of the Institutional Animal Care and Use Committee. Knock-in transgenic mice expressing *Cre* recombinase from the parvalbumin (*PV*) locus (Hippenmeyer et al., 2005; JAX Stock 008069) were crossed with mice that conditionally expressed the GECI GCaMP6s (*PV-cre/+;GCaMP/+*; JAX Stock 024106) from the *ROSA26* locus (Chen et al., 2013) to enable optical recording of proprioceptive sensory neuron activity within the DRG. PV is a calcium-binding protein expressed in subsets of neurons in the brain (Cowan et al., 1990; Schwaller et al., 2002), spinal cord (Zacharova et al., 2009), and DRG (Honda, 1995; de Nooij et al., 2013). In the 5th lumbar (L5) DRG of a mouse, >85% of PV+ neurons are proprioceptors, and the remainder are low-threshold cutaneous mechanoreceptors (de Nooij et al., 2013). To minimize the probability of imaging PV+ mechanoreceptors, low intensity electrical currents (<10 µA; Sdrulla et al., 2015) were used. While living neurons cannot be definitively classified as proprioceptors without employing techniques to assess their functional connectivity, the use of low-intensity electrical currents on a population of neurons that is already >85% proprioceptors ensured that the vast majority of cells analyzed in this study were proprioceptors. A total of 11 female and 10 male adult (more than two months) *PV-cre/+;GCaMP/+* mice were used in this study.

### *Ex vivo* sciatic nerve-DRG preparation

Animals were anesthetized via intraperitoneal injection of Euthasol (≥270 mg/kg, i.p.; Virbac), and transcardially perfused with 5 ml of ice-cold oxygenated (95% O_2_; 5% CO_2_) artificial CSF (ACSF) containing the following: 127 mM NaCl, 1.9 mM KCl, 1.2 mM KH_2_PO_4_, 1 mM MgSO_4_7H_2_O, 26 mM NaHCO_3_, 16.9 mM D(+)-glucose monohydrate, and 2 mM CaCl_2_. Next, animals were decapitated, and the spinal column and right hindlimb were dissected out and submerged in a chamber filled with recirculating ACSF (16–18°C). A dorsal laminectomy was performed, and the dura was removed to expose DRGs to circulating ACSF.

**Figure 1.**
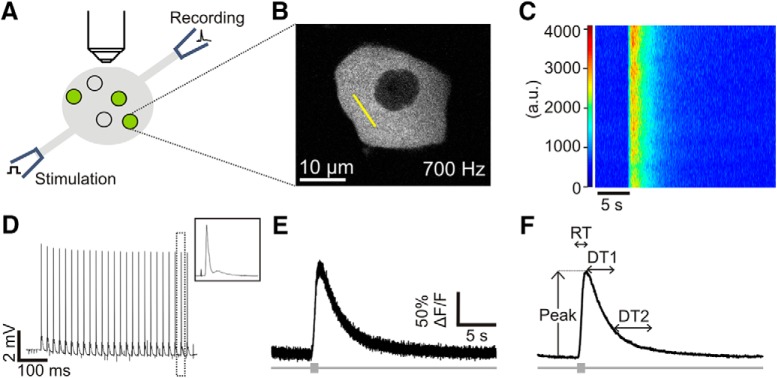
Approach for calcium imaging of proprioceptors in the intact DRG. ***A***, Schematic of the imaging preparation. Electrical stimulation of the sciatic nerve evoked action potentials in peripheral sensory neurons. Compound action potentials were recorded via a suction electrode on the proximal dorsal root. PV+ sensory neurons, consisting mainly of proprioceptors but also some mechanoreceptors, expressed GCaMP6s using a Cre-*lox* strategy. Two-photon calcium imaging data were acquired from single neurons in the intact lumbar 5 (L5) DRG. ***B***, A representative image of a GCaMP6s-expressing DRG neuron responding to electrical stimulation. Yellow line indicates the region chosen for line scan analysis (at ∼700-Hz scanning rate). ***C***, Heat map of the electrically evoked change in fluorescence across the line scan (oriented vertically) over time (horizontal axis). Optical recording begins 5 s before stimulus is delivered to establish pre-stimulus resting fluorescence. ***D***, Compound action potentials recorded from the L5 dorsal root during the stimulus train. Action potentials are observed with every electrical pulse (0.1-ms pulses at 50 Hz for 0.5 s). Amplitude of the compound action potential remains stable throughout the stimulus. Area within dotted box is enlarged in the box on the upper right corner. ***E***, Representative calcium transient evoked by electrical stimulation (box on gray bar). The average fluorescence across the length of the line scan at each time point composes the raw calcium transient. ***F***, A smoothing algorithm removes high frequency signal fluctuations. Analysis scripts quantify four parameters of the transient: peak amplitude (peak), RT, decay constant (DT1), and recovery time (DT2). For descriptions of each parameter, see Results.

The right sciatic nerve was transected mid-thigh and dissected proximally to the level of the dorsal roots. The final preparation contained only the right sciatic nerve connected to the L5 DRG and L5 dorsal root (Fig. 1*A*). To optimize imaging quality by reducing the amount of light-scattering surfaces, a collagen-breaking solution (12.5% 10 mg/ml collagenase Type I; Sigma-Aldrich C0130, 12.5% thermolysin; Sigma-Aldrich P1512 1000 U/ml, 75% H_2_0) was applied to the surface of the L5 DRG using a small-tipped glass pipette. After the enzyme solution incubated for 10–15 min, residual connective tissue was dissected from the DRG surface. Finally, glass pipette suction electrodes (A&M Systems) were fire-polished and sized to fit the sciatic nerve and L5 dorsal root. After the dissection was completed, the temperature of the recirculating ACSF was allowed to warm to room temperature (22–24°C) for 1 h before calcium imaging began.

### Calcium imaging

Optical recordings of PV+ neuron calcium transients evoked by electrical stimulation were made in the L5 DRG via two-photon laser line scans (Olympus FV1000-MPE; 25× 1.05 NA objective, 920-nm excitation wavelength). At the beginning of each experiment, the DRG was examined under epifluorescence to identify cells that were strongly fluorescent in the absence of stimulation; these cells (average, four cells; range, 0–12 cells; *n* = 21 animals) were likely damaged in the dissection and were excluded from analysis. Next, the sciatic nerve was stimulated with low current pulse trains (0.1-ms pulse at 50 Hz for 1 s followed by 2-s rest intervals, stimulation current < 10 µA; A360 stimulus isolation unit; WPI) and responding cells were identified by visual inspection using epifluorescence. More cells responded to stimulation than could be imaged over the course of one experiment, so the following procedures were employed to ensure systematic sampling of cells. The user began sampling neurons at the highest, most shallow plane of the DRG. All responsive cells in the same focal plane were imaged, one at a time, before moving to a deeper focal plane to image the next group of responsive cells.

Line scans of a consistent length (9.3 µm, 76 pixels in our system; Fig. 1*B*) were used to maximize the sampling frequency and were drawn across the brightest part of the cytosol, excluding the nucleus. Laser intensity and detector sensitivity [photomultiplier tube (PMT) voltage] were held constant between and within experiments. The line was scanned 20,000 times (total time, 28.16 s; sampling frequency >700 Hz) to capture the entire time course of a calcium transient evoked by sciatic nerve stimulation (Fig. 1*C*). Pre-stimulus resting fluorescence was recorded for 5 s before a 0.5-s pulse train (0.1-ms pulses at 50 Hz) was delivered to the sciatic nerve at supramaximal intensity (0.6 mA) to produce 25 action potentials. Control experiments determined a train of 25 action potentials at 50 Hz consistently elicited calcium transients of reliable size and shape.

Extracellular recordings of compound action potentials in the L5 dorsal root were made to confirm the consistency of the nerve stimulation (EX4-400 Quad differential amplifier, low cut filter: 2 Hz, high cut filter: 10 kHz, gain: 1000×; Dagan Corp.; digitized using Clampex, version 10.7; Fig. 1*D*). A custom script allowed the stimulation software (Clampex) to trigger and control the scanning software (Olympus Fluoview, version 4.0b). Optical recordings for each cell were repeated three times and averaged and analyzed offline.

### Calcium concentration and electrical activity manipulations

Alterations to the stimulation protocol and bath calcium concentration examined the impact of these parameters on the properties of GECI transients. To manipulate electrical activity, the duration of the sciatic nerve pulse train was varied to evoke different numbers of action potentials (1–100 action potentials). The inter-pulse interval was held constant to deliver stimuli at 50 Hz, but the number of pulses in a train and therefore overall stimulation duration, was altered. These stimulation parameters were repeated in the same cells for three different ACSF conditions. First, cells were imaged in normal Ca^2+^ ACSF (2 mM), and then in high Ca^2+^ ACSF (4 mM). Lastly, cells were imaged in ACSF where no calcium was added. When switching bath conditions, a reservoir collected and removed re-circulating ACSF from previous baths to reduce mixing of bath concentrations. Residual calcium from previous ACSF solutions was not chelated, so relatively low calcium concentrations likely remained in the no-added calcium ACSF condition.

During two of these experiments, in normal Ca^2+^ ACSF, the frequency of electrical stimulation was manipulated to determine whether individual action potentials could be identified in GCaMP6s transients with low frequency stimulation and whether GCaMP6s transients reached a maximal size with high frequency stimulation. Cells were stimulated for 1 s at 1 Hz and then for 0.5 s at the following frequencies: 5, 10, 25, 50, 75, 100, 150, 200, 250, and 300 Hz. Each cell was given frequency stimulations in a random order, intentionally switching back and forth from a high frequency stimulation (>100 Hz) to a low frequency stimulation (<100 Hz), until all frequencies had been tested, before moving on to the next cell.

### Calcium pump manipulation

Another subset of experiments tested the effect of thapsigargin (TG; Sigma-Aldrich), a selective non-competitive inhibitor of SERCA, on PV+ calcium transients. TG was diluted in dimethyl sulfoxide (DMSO) before being added to bath (1 µM TG, 0.1% DMSO final concentrations). In vehicle experiments, DMSO (0.1%) was added to the bath. Before adding TG or vehicle, baseline transients were acquired in standard ACSF. The same cells were re-imaged 30 min after TG or vehicle was added to the recirculating ACSF. Three animals (two males, one female) were used for vehicle experiments, and two animals (one male, one female) were used for TG experiments.

In two additional experiments (two males), bath pH was manipulated to investigate the effect of decreased PMCA activity on GECI calcium transients in PV+ neurons. In rat DRG neurons, PMCA is non-selectively and reversibly blocked by increasing extracellular pH to 8.8 (Benham et al., 1992). For these experiments, Tyrode solution was substituted for ACSF, containing the following: 140 mM NaCl, 4 mM KCl, 2 mM CaCl_2_, 2 mM MgCl_2_, 10 mM HEPES, and 10 mM D(+)-glucose monohydrate, pH 7.3. Cells were imaged at baseline in Tyrode solution, a second time in a Tyrode solution containing Trizma (10 mM; pH 8.8) instead of HEPES, and a third time in the original Tyrode solution with HEPES (pH 7.3).

### Data analysis

Line scan time series data acquired in Fluoview were analyzed with scripts in MATLAB (R2015a). Transient responses were first processed using a Butterworth filter in the “designfilt” function in MATLAB, followed by an iterative smoothing process (Yaksi and Friedrich, 2006) to reduce noise (compare Fig. 1*E*, *F*). The rising phase of the transient was fit using a polynomial function, while a second-order exponential decay function best fit the decay. From these equations, the slope of the rising phase and a decay coefficient [decay time 1 (DT1)] were determined for each transient. Transient RT, peak amplitude, and the time required for the transient to decay from DT1 to 10% of resting fluorescence (DT2) were also extracted.

Linear regressions were performed in SPSS (Statistics 23). Five one-way mixed effects ANOVAs, implemented with SAS (version 9.4), were used to compare the fixed effect of sex, as well as the random effects of litter and animal on the calcium-transient properties measured: resting fluorescence, peak amplitude, RT, DT1, and DT2. Values for peak amplitude underwent a natural log transformation to meet the mixed effects model criteria of normality and homogeneity of variances. The Freedman–Diaconis rule was used to determine the number of bins for each histogram (Freedman and Diaconis, 1981). To determine whether the calcium transient parameters of PV+ cells were distributed unimodally or multimodally, the dip test of unimodality (Hartigan and Hartigan, 1985) examined histogram data for statistically significant “dips,” in distributions (https://CRAN.R-project.org/package=diptest; R package version 0.75-7, 2015). Paired *t* tests compared baseline transient parameters to those after vehicle or drug. Corrections for multiple comparisons were made using a step-down Bonferroni multiple comparison procedure (Holm, 1979), which adjusted α to 0.005 for linear regressions (α = 0.05/11 regressions), 0.01 for ANOVAs (α = 0.05/5), and 0.01 for paired *t* tests (α = 0.05/5 paired *t* tests for each drug/vehicle). A power analysis was performed using an open-access power calculator created by Sealed Envelope (Kim and Seo, 2013).

## Results

### Calcium imaging data acquisition and analysis

Using transgenic mice that expressed GCaMP6s in PV+ neurons, we visualized proprioceptive neurons in an *ex vivo* preparation of the L5 DRG. Dozens of cell somas fluoresced in response to electrical stimulation of the sciatic nerve, and 10 ± 2 cells (mean ± SD, *n* = 18) were imaged each experiment. Cells were chosen for imaging using a systematic approach to ensure a random sample of cells was imaged in each experiment (for details, see Materials and Methods). In this way, each experiment captured the calcium dynamics of cells with a range of fluorescent intensities rather than only the most brightly fluorescing cells. Data from individual cells were collected using smaller regions of interest via line scans, which enhanced temporal resolution enough to reliably record calcium transients (Fig. 1). This approach to optical recording, as well as offline data analysis and noise reduction steps, produced calcium transients with quantifiable characteristics from each imaged cell.

### GECI calcium transient variables

To test the hypothesis that GECI calcium transients can produce meaningful information about the calcium dynamics of a cell, calcium transients were analyzed by variables described in studies that used synthetic dye indicators (Gemes et al., 2011; Scheff et al., 2013; Fig. 1*F*). Parameters that describe the rising phase of the transient included peak amplitude, RT, and slope. Peak amplitude represents the point along the transient in which the change in fluorescence is maximal. The time interval between the onset of electrical stimulus and the peak amplitude was defined as RT, and slope was calculated from the polynomial fit of the rising phase of the transient. Two parameters that describe the transient decay were also calculated. DT1 is the negative inverse of the decay coefficient produced by the second-order exponential decay function, and represents the time needed for the peak amplitude to decay 63%. Previous studies have used T_90_, or 90% recovery time, to measure the time necessary for calcium transients to recover to pre-stimulus resting levels (Scheff et al., 2013). However, preliminary analyses of the present data showed that T_90_ could be predicted by DT1, as DT1 accounted for 70% of T_90_ (63% of 90% recovery). Rather than reporting values for a variable that would be redundant in this study, DT1 was subtracted from T_90_, creating the new variable DT2. Thus, DT2 represents residual recovery time; the additional time it takes for the calcium transient to recover to resting levels after DT1. DT2 was calculated by subtracting DT1 from the time required for the transient to return to 10% above resting levels.

In addition to the five calcium transient variables described above, the average pixel intensity during the first 100 ms of the line scan, reported in the arbitrary units of the PMT detector, was measured to estimate resting fluorescence. This provides an indirect measure of GCaMP6s expression; cells with higher fluorescence at rest are likely to have more fluorescent molecules, i.e., GCaMP6s.

**Figure 2.**
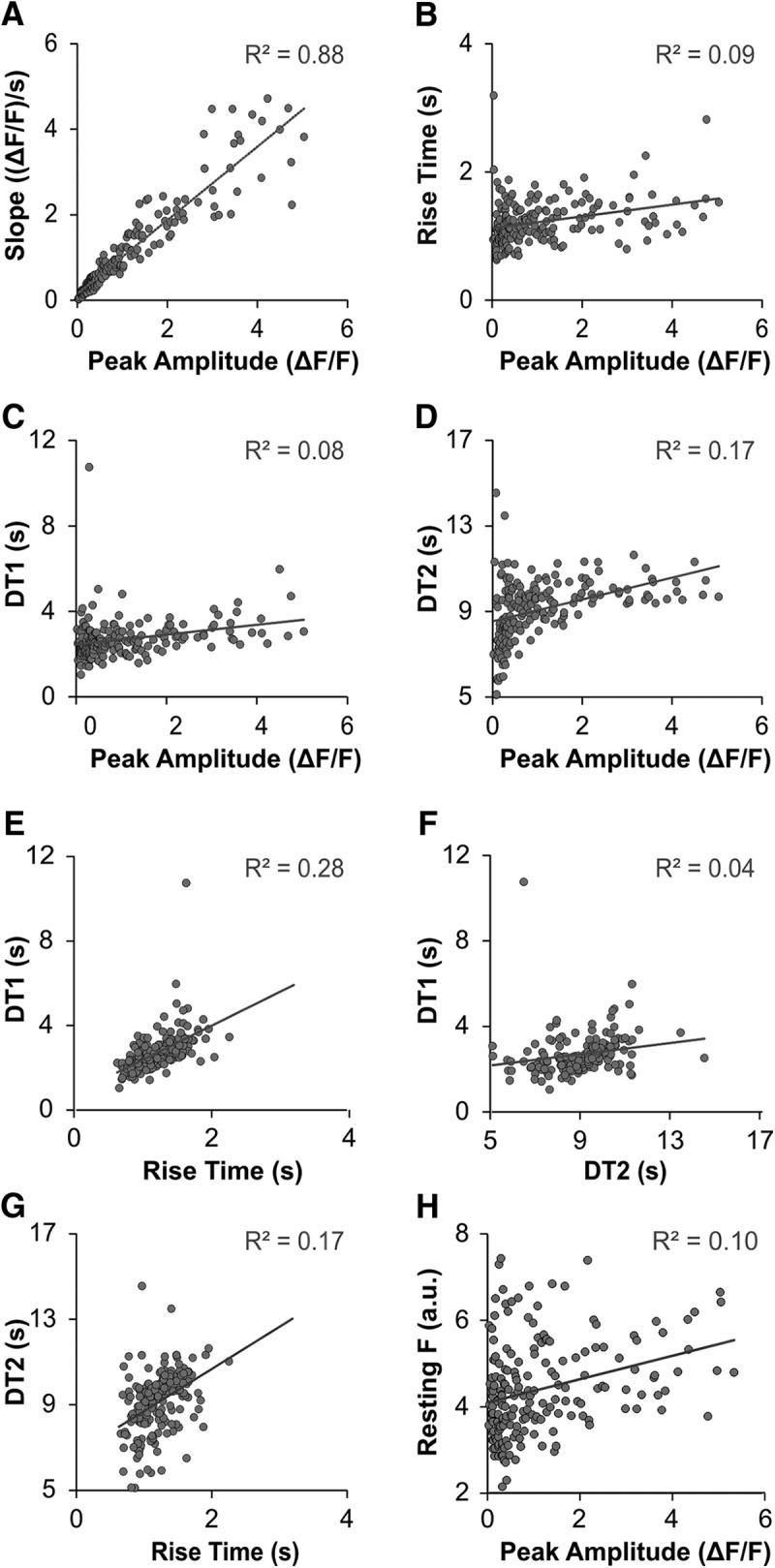
Linear regression analysis of calcium transient variables. ***A–H***, Other than slope and peak amplitude (*R* ^2^ = 0.88), only a fraction of variability in one variable was predicted by other variables (*R* ^2^ range, 0.04–0.28). DT1, the time required to decay 63% from the peak fluorescence; DT2, the residual recovery time necessary to recover from DT1 to 90% of resting values. Resting F, fluorescence of the cell at rest, plotted as PMT a.u./100.

Linear regressions compared resting fluorescence and five calcium transient variables to each other to determine the extent one parameter would predict another and was therefore under control of similar calcium-regulating mechanisms. The variability in peak amplitude was significantly predicted by rising slope variability (*R* ^2^ = 0.88, β = 1.02, *t*_(186)_ = 36.33, *p* < 0.0001; Fig. 2*A*). Peak amplitude significantly predicted the other four variables (*p* < 0.0001), but with smaller *R* ^2^ values demonstrating that peak amplitude variability predicted only a small portion of RT (9%, *R* ^2^ = 0.09, β = 1.02, *t*_(186)_ = 4.39; Fig. 2*B*), DT1 (8%, *R* ^2^ = 0.08, β = 0.37, *t*_(184)_ = 4.05; Fig. 2*C*), DT2 (17%, *R* ^2^ = 0.17, β = 0.33, *t*_(178)_ = 5.95; Fig. 2*D*), and resting fluorescence variability (10%, *R* ^2^ = 0.10, β = 0.003, *t*_(186)_ = 4.41; Fig. 2*H*). Similarly, the variability in other variables were predicted by each other with small *R* ^2^ values (<0.30): DT1 versus RT (28%, *R* ^2^ = 0.28, β = 1.38, *t*_(184)_ = 8.48, *p* < 0.0001), DT1 versus DT2 (4%, *R* ^2^ = 0.04, β = 0.13, *t*_(178)_ = 2.78, *p* = 0.006), DT2 versus RT (17%, *R* ^2^ = 0.17, β = 1.67, *t*_(178)_ = 5.93, *p* < 0.0001; Fig. 2*E–G*). These findings indicated that slope could be removed from further analyses as slope values are analogous to peak amplitude. On the other hand, it was important to maintain the following variables of interest in further analyses because they vary independently from each other for the majority of cells: resting fluorescence, peak amplitude, RT, DT1, and DT2.

### Description of PV+ calcium transients

**Figure 3.**
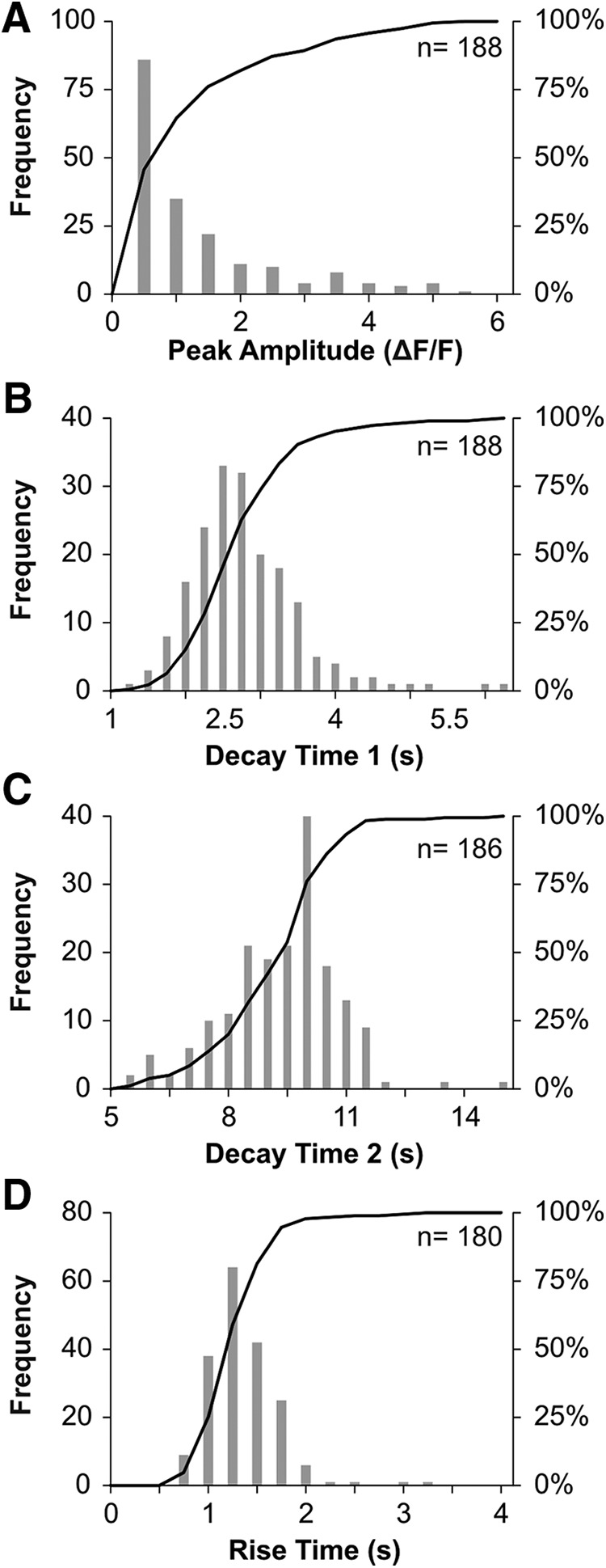
Histograms illustrating distributions of four parameters quantified from each cell imaged. ***A–D***, Frequency (gray boxes) and cumulative (black line) histograms for each parameter. Sample size for each parameter is given in the upper right-hand corner. Distributions do not show evidence of multimodality.

The characteristics of calcium transients from a large sample of PV+ neurons (188 cells from 20 animals) were pooled and analyzed at a population level to determine whether subpopulations within the PV-containing DRG subclass could be identified. Dip tests for unimodality determined there was no bimodality or multimodality in any of the distributions (resting fluorescence *p* = 0.99, peak *p* = 0.95, RT *p* = 0.61, DT1 *p* = 0.92, DT2 *p* = 0.99). While unimodally distributed, the peak amplitudes of the electrically evoked calcium transients displayed considerable variability (Fig. 3*A*). The median maximal change in fluorescence was an increase of 60% over rest (%ΔF/F) but could go as high 504%, resulting in a distribution of peak amplitudes that was skewed toward larger amplitudes. The interquartile range for peak amplitudes was 27–144%, and the range was 502%. In contrast to peak amplitude, other parameters of PV+ transients (RT, decay parameters, and resting fluorescence) demonstrated less variability and were more normally distributed (Fig. 3*B–D*). The average RT was 1.22 ± 0.35 s (mean ± SD). The average DT1 was 2.62 ± 0.91 s, and the average DT2 was 9.07 ± 1.63 s. The average resting fluorescence was 438 ± 108 a.u.

**Figure 4.**
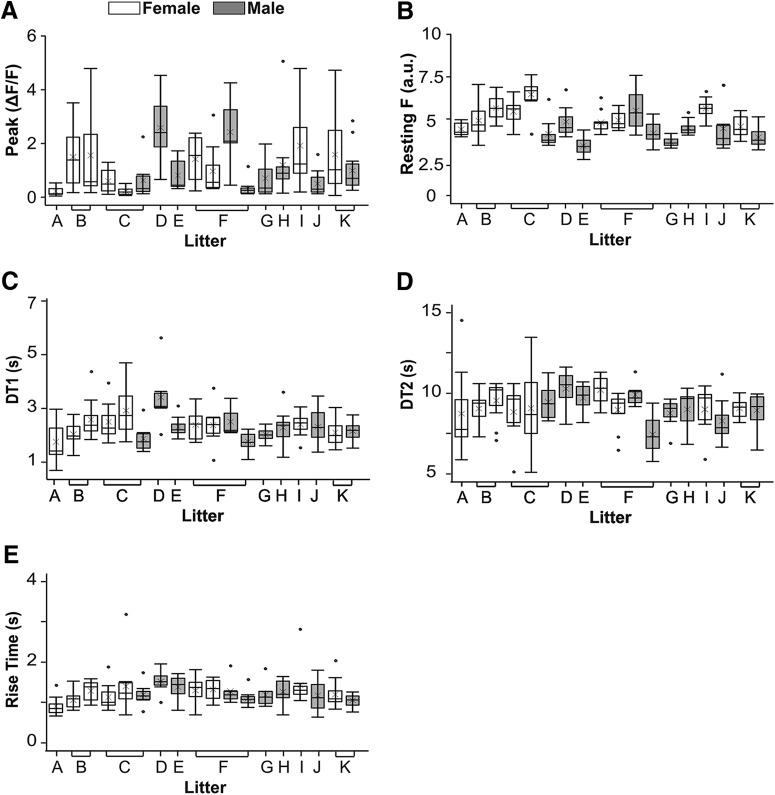
Variation in calcium transients between cells, animals, and sexes. Each box plot represents data from 7–15 cells from one animal. Animals are grouped together by litter (labeled A-K). Shaded boxes indicate males and empty boxes indicate females. For each box plot: lower bar = first quartile, box bottom = 2nd quartile, horizontal line = median, x = mean, box top = 3rd quartile, top bar = 4th quartile, circles outside plot = data more or less than 1.5 times the interquartile range (box). ***A***, Tall boxes and bars demonstrate that the peak amplitudes of calcium transients varied from cell to cell. Differing medians, means, and box heights show that peak amplitudes also varied from animal to animal. There are no differences between males and females or litters in the distribution or variability of peak amplitudes. ***B***, In contrast to peak amplitude, resting fluorescence (Resting F; plotted as PMT a.u./100) is more consistent across cells, animals, and sexes. ***C–E***, RT, DT1, and DT2 also demonstrate less variability between cells and animals than peak amplitude.

The mixed-effects models revealed no significant differences between males and females for peak amplitude (*F*_(1,18)_ = 0.08, *p* = 0.77), RT (*F*_(1,18)_ = 0.00, *p* = 0.95), DT1 (*F*_(1,18)_ = 0.39, *p* = 0.54), or DT2 (*F*_(1,18)_ = 0.25, *p* = 0.62; Table 1). The model did identify a significant difference between male and female resting fluorescence values: females 486 ± 87 versus males 398 ± 88 (mean ± SD a.u.), *F*_(1,18)_ = 9.24, *p* = 0.007. Fluorescent intensities are reported in the arbitrary units of the imaging system and the mean values for males and females are only 9.7% and 11.8%, respectively, of the maximum intensity values allowed in the system (4095 a.u.), suggesting these values likely do not represent biologically relevant differences. As observed in the aggregate data, peak amplitude variability between animals was greater than for the other three transient properties (Fig. 4). Highly variable peak amplitudes were also evident within the same animal. Within the same experiment, some cells showed minimal changes in fluorescence (12%ΔF/F) while other cells fluoresced 8× more (504%ΔF/F) than the median change in fluorescence (60%ΔF/F). The differences between cells occurred even among cells within the same focal plane of the DRG. In summary, there was a high degree of variability in peak amplitudes between cells, but this degree of variability was consistent across animals, litters, and sexes.

**Table 1.** Analysis of sex differences in transient parameters

	Main effect *p*	Female (*n* = 9)	Male (*n* = 9)
Peak (%ΔF/F)	0.77	55 [27–145] (97)	60 [24–135] (82)
RT (s)	0.95	1.22 ± 0.39 (97)	1.22 ± 0.30 (82)
DT 1 (s)	0.54	2.74 ± 1.08 (95)	2.59 ± 0.68 (82)
DT 2 (s)	0.62	9.15 ± 1.51 (92)	8.96 ± 1.37 (79)

Median [IQR] or mean ± SD. Parentheses indicate number of animals (headings) or cells (table). One-way mixed effects ANOVAs compared the transient properties of males and females and found no significant differences (*p* > 0.01) between sexes for these four properties.

### Effects of alterations to electrical activity and external calcium concentrations

**Figure 5.**
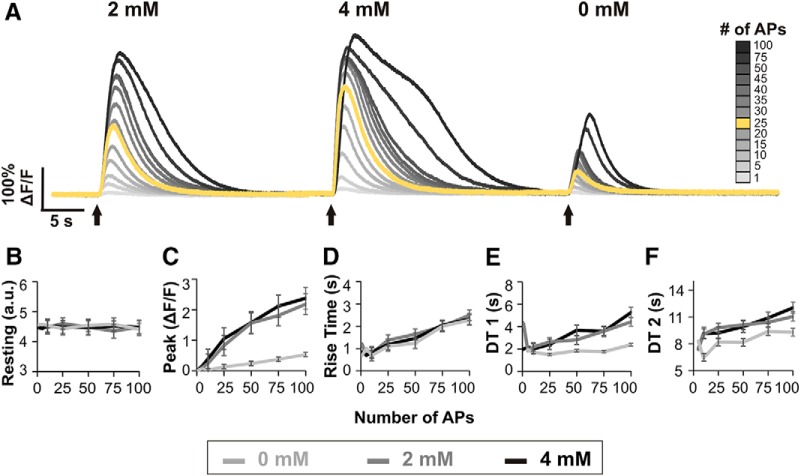
Effects of alterations in electrical activity and extracellular calcium concentrations on calcium transients. ***A***, Example calcium transients from one cell in three different bath conditions: ACSF with normal (2 mM), high (4 mM), and low calcium (no calcium added). In addition to manipulating extracellular calcium concentrations by changing bath calcium, intracellular calcium concentrations were manipulated by stimulating different numbers of action potentials (APs). For reference, the standard stimulation of 25 APs used elsewhere in this study is shown in yellow. ***B–F***, Plots of transient parameter changes with varying bath calcium concentrations and AP numbers. Each dot represents the average of six cells. Error bars represent the SEM. When less than three cells produced measurable transient parameters, error bars were not possible and thus omitted, i.e., at one and five APs. Resting fluorescence is plotted as PMT a.u./100.

We next explored whether the measured parameters of GCaMP6s calcium transients could provide meaningful information about the electrical activity and calcium dynamics of a cell. First, neuronal electrical activity and associated calcium fluxes were altered by evoking different numbers of action potentials and by varying the calcium concentration of the bath. As intracellular and extracellular calcium concentrations were manipulated in this way, the properties of calcium transients changed (Fig. 5*A*). Dorsal root recordings confirmed the stimulation protocol evoked the appropriate number of action potentials (data not shown; but see Fig. 1*D* for example of action potentials evoked by 50-Hz stimulation for 0.5 s).

The stimulus parameters used elsewhere in this study (25 action potentials at 50 Hz), elicited transients that fell within the middle of each cell’s range of transient sizes (Fig. 5*A*, yellow line). Stimulation with fewer than five action potentials elicited no measurable transients in five of six cells analyzed. Stimulation with five or more action potentials evoked transients in all six cells studied. In contrast, transients elicited by >50 action potentials demonstrated shapes that varied from other transients, i.e., a plateau during the decay phase or a change in slope during the rise phase.

Resting fluorescence remained stable for each cell regardless of the number of action potentials elicited or the calcium concentration of the bath (Fig. 5*B*). The peak amplitude of calcium transients increased with longer trains of action potentials (Fig. 5*C*). Extracellular calcium also had a clear effect on peak amplitude. Transient peaks were smallest in the low-calcium bath, larger in normal calcium, and largest in high calcium. A wide range of response amplitudes could be observed in the same cell, for example from a 4%ΔF/F change from rest (no calcium added bath, 1 action potential) to 400%ΔF/F (high calcium bath, 100 action potentials).

The RT of calcium transients rose consistently with increasing numbers of action potentials, i.e., increased intracellular calcium fluctuations, but did not show a clear relationship with extracellular calcium, as RT for all three bath conditions overlapped (Fig. 5*D*). DT1 was altered by both changes in intracellular and extracellular calcium concentrations. Elevating the number of action potentials increased DT1 in both normal and high calcium, but DT1 values remained relatively stable regardless of action potential number in no calcium added ACSF (Fig. 5*E*). Residual decay time (DT2) was affected by both changes in intracellular and external calcium. As the number of action potentials increased from 5 to 100, DT2 increased by ∼1.8 s after correcting for differences in stimulus duration. DT2 times were similar in normal and high calcium baths but were shorter in low calcium conditions (Fig. 5*F*).

**Figure 6.**
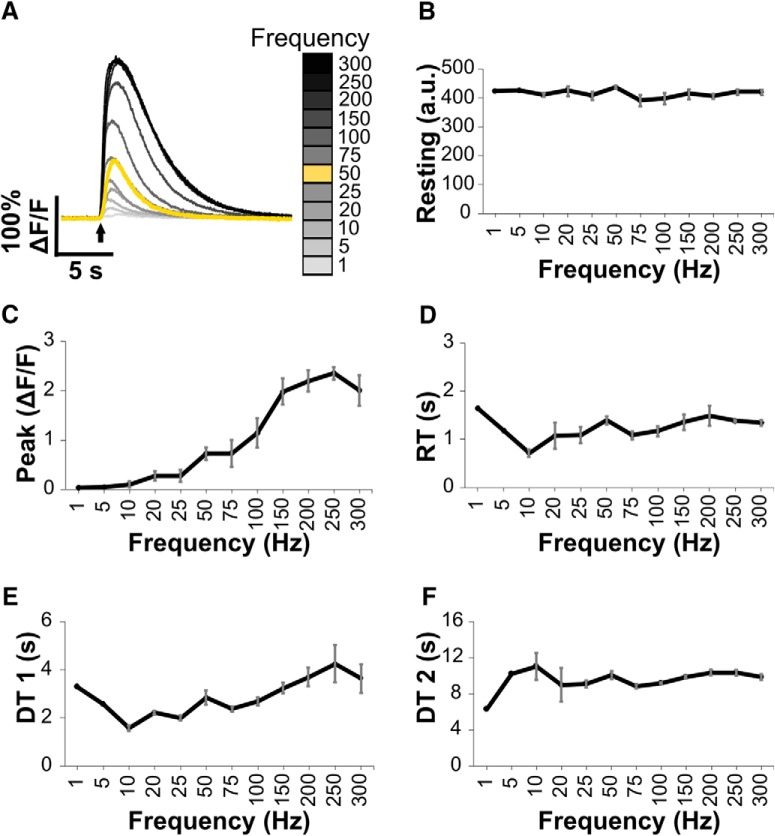
Calcium transients evoked by nerve stimulation at various frequencies. ***A***, Example transients from one cell following stimulation for 0.5 s at frequencies from 1 to 300 Hz. For reference, the standard stimulation of 25 action potentials used elsewhere in this study is shown in yellow. ***B***, Resting fluorescence measured in arbitrary units does not change with increasing stimulation frequencies. ***C–F***, Plots of transient parameter changes with varying stimulation frequencies. Error bars indicate SEM for responses evoked by stimulation frequencies of 10 Hz and higher.

In addition to varying the number of action potentials evoked at a set frequency, stimulation trains at multiple frequencies (ranging from 1 to 300 Hz) were used as a second means to alter PV+ neuron electrical activity (Fig. 6). Analysis of dorsal root recordings suggested stimulation frequencies up to 75 Hz elicited compound action potentials with consistent amplitudes. However, at higher frequencies compound action potential amplitudes decreased during the stimulation train, suggesting not all activated neurons could maintain these high firing rates (data not shown). Resting fluorescence remained stable for each cell across the range of stimulation frequencies, suggesting efficient recovery of intracellular free calcium concentrations between stimulation episodes (Fig. 6*B*). With 1-Hz stimulation, calcium transients were small (2–6%ΔF/F) and noisy in three cells and undetectable in one cell. From 5 to 10 Hz, one cell produced reliable transients (peak amplitudes between 15% and 29%ΔF/F), while the transients for the other three cells remained small and noisy. All four cells produced reliable transients (peak amplitudes >5%ΔF/F) by 20 Hz, and peak amplitudes grew higher with increased stimulation frequency up to 150 Hz and then plateaued. DT1 lengthened with increased stimulation frequency up to 250 Hz, and then plateaued. RT and DT2 were unaffected by changes in firing frequency.

### Effects of blocking calcium pumps

**Figure 7.**
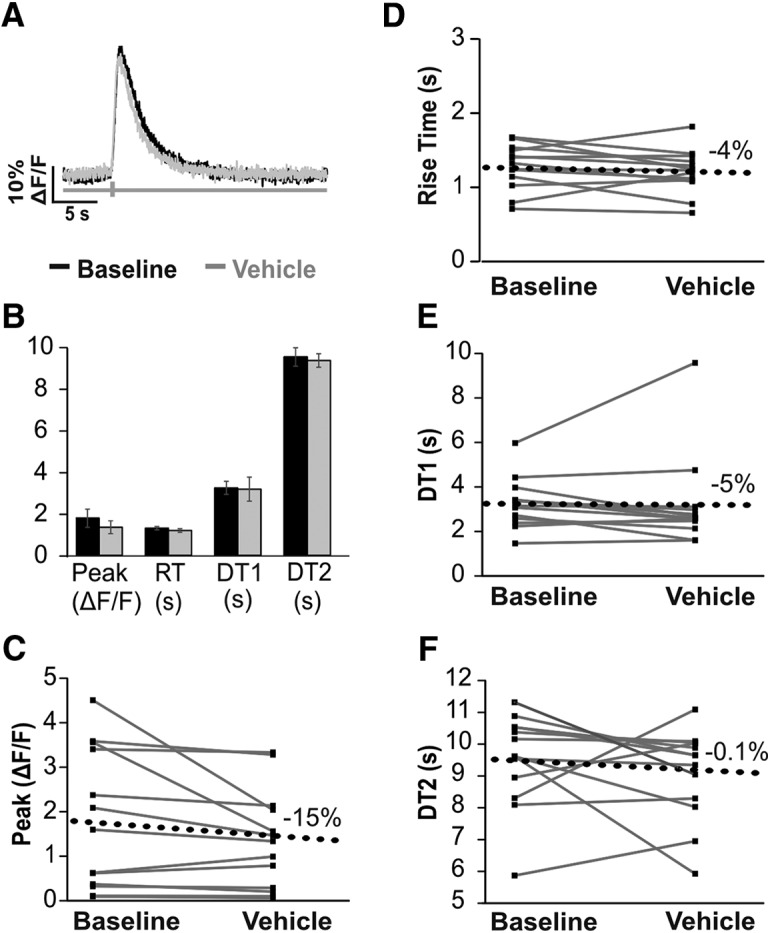
GCaMP6s calcium transients do not significantly change with repeat imaging. ***A***, Example calcium transients from one cell imaged at baseline (black) and 30 min after vehicle (ACSF with 0.1% DMSO, gray). ***B***, Mean values (±SEM) from 13 cells imaged from three animals at baseline and after vehicle. Paired *t* tests found no significant differences after addition of vehicle were detected. ***C–F***, Baseline and vehicle values for 13 cells (gray lines). Black dotted line denotes the average change from baseline for each parameter.

The suitability of GCAMP6s to provide information about the function of specific calcium regulatory machinery was assessed by imaging cells before and after the addition of compounds to block the function of two calcium pumps. SERCA activity was inhibited by using TG, and PMCA activity was diminished by elevating the bath pH. We first examined the stability of calcium responses over time by imaging cells before and after addition of the vehicle (Fig. 7). Resting fluorescence remained the same after the addition of vehicle [*n* = 13, mean ± SD (a.u.)]; baseline 442 ± 92; vehicle 441 ± 104; average % change from baseline = 0.4%, paired *t*_(12)_ = 2.18, *p* = 0.97. For some cells, the peak amplitude increased over time and for other cells the peak amplitude decreased so that the average change in peak amplitude from baseline was –15% (*n* = 13, median [IQR] (%ΔF/F); baseline 160 [35–348]; vehicle 133 [25–209]). Similarly, RT varied up and down resulting in an average change of –4% [*n* = 13, mean ± SD (s); baseline 1.27 ± 0.30; vehicle 1.20 ± 0.30]. DT1 values change –5% on average [*n* = 13, mean ± SD (s); baseline 3.24 ± 1.12; vehicle 3.18 ± 2.07]. Average DT2 values remained almost identical [*n* = 12, mean ± SD (s); baseline 9.52 ± 1.52; vehicle 9.34 ± 1.12]. Thus, on repeat imaging, the calcium transients for a group of cells will differ <15% on average from their original values. Neither peak amplitude, RT, DT1, nor DT2 significantly differed from measurements at baseline to measurements after vehicle: peak amplitude (paired *t*_(12)_ = 2.18, *p* = 0.08), RT (paired *t*_(12)_ = 2.18, *p* = 0.31), DT1 (paired *t*_(12)_ = 2.18, *p* = 0.87), and DT2 (paired *t*_(11)_ = 2.20, *p* = 0.67). Although the *p* value for peak amplitude appears to approach significance, after correcting for five separate comparisons, the threshold for significance is *p* < 0.01. Furthermore, a power analysis revealed a sample size of 265 cells would be required for peak amplitude to significantly differ from baseline.

**Figure 8.**
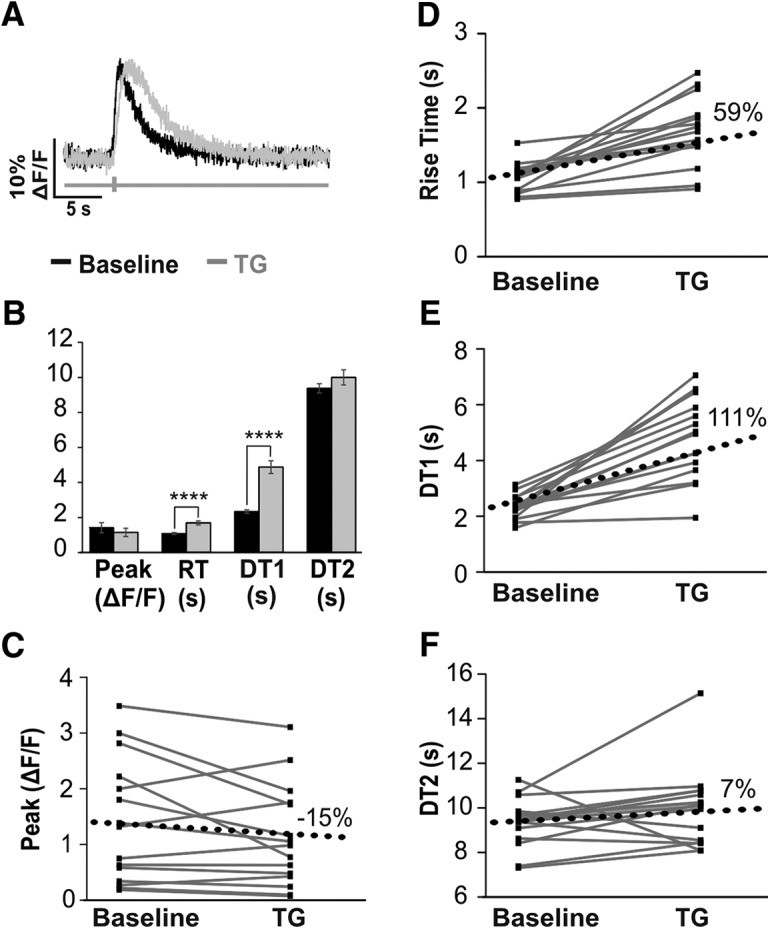
GCaMP6s calcium transients provide insights into the activity of SERCA. ***A***, Example calcium transient from a cell at baseline recording (black) and after the addition of the SERCA-inhibitor, TG (gray). ***B***, Mean values (±SEM) from 16 cells from two animals imaged at baseline and after addition of TG. Paired *t* tests revealed significant changes, which are indicated (****p < 0.0001). ***C–F***, Plots for transient parameters at baseline and after the addition of TG for 16 cells. Black dotted line denotes the average change from baseline for each parameter.

SERCA pumps were blocked using the selective inhibitor TG. The average change in resting fluorescence after the addition of TG was –5% [*n* = 16, mean ± SD (a.u.); baseline 441 ± 112; TG 462 ± 106, paired *t*_(15)_ = 2.13, *p* = 0.09]. The average change in peak amplitude after the addition of TG was –15% (*n* = 15, median [IQR] (%ΔF/F); baseline 132 [34–222]; TG 98 [43–175], paired *t*_(14)_ = 2.14, *p* = 0.09; Fig. 8). RT increased on average by 59% [*n* = 15, mean ± SD (s); baseline 1.07 ± 0.20; TG 1.68 ± 0.46]. DT1 increased on average by 111% [*n* = 16, mean ± SD (s); baseline 2.32 ± 0.41; TG 4.86 ± 1.46]. The average change of DT2 was 7% [*n* = 16, mean ± SD (s); baseline 9.35 ± 1.06; TG 9.98 ± 1.72, paired *t*_(15)_ = 2.13, *p* = 0.13]. Unlike resting fluorescence, peak amplitude, or DT2, values for RT and DT1 significantly differed after the addition of TG (RT paired *t*_(14)_ = 2.14 paired *t*_(14)_ = 2.14, *p* < 0.0001, DT1 paired *t*_(15)_ = 2.13, *p* < 0.0001). RT and DT1 were also the only variables where cells all changed in the same direction. In summary, blocking SERCA pumps significantly increased RT and DT1, but did not affect resting fluorescence, peak amplitude, or DT2.

**Figure 9.**
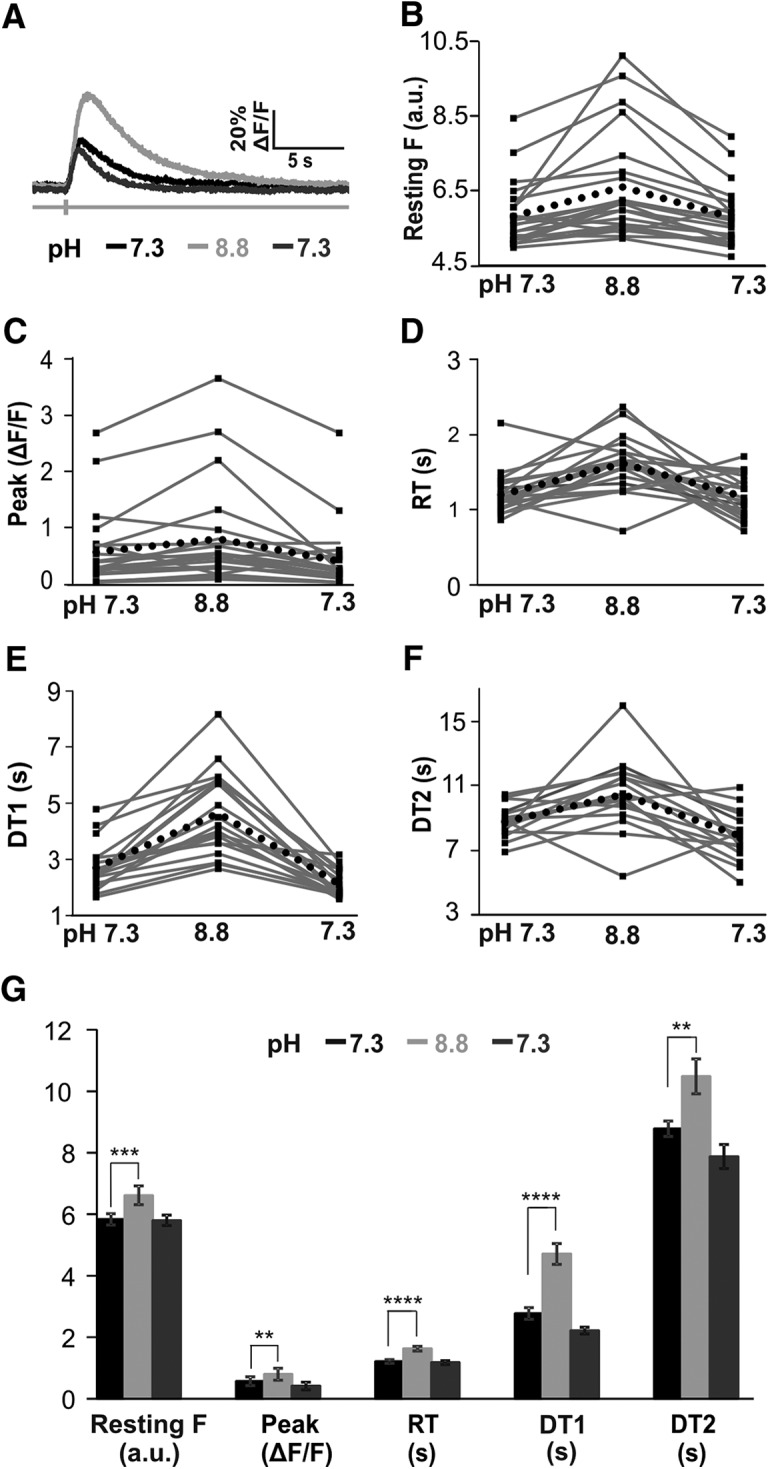
Inhibiting PMCA activity by elevating bath pH to 8.8 increases the calcium transients of PV+ neurons. Calcium transients are restored when bath pH is returned to 7.3. ***A***, Example calcium transients from a cell in normal solution (pH 7.3, black), in elevated bath pH conditions (pH 8.8, light gray), and after returning to normal bath pH (pH 7.3, darker gray). ***B–F***, Profiles of transient parameter changes in 22 cells from normal bath pH values (7.3) to elevated pH (8.8) and after returning to normal pH (7.3). Black dotted line denotes the average change from baseline for each parameter. ***G***, Mean values (±SEM) from 22 cells from two animals imaged at baseline and elevated pH. Paired *t* tests revealed significant changes, which are indicated (**p < 0.01, ***p < 0.001, ****p < 0.0001).

Finally, the effect of PMCA activity on GECI calcium transients was assessed by inhibiting PMCA with increased bath pH. When PMCA was inhibited, resting fluorescence and all four transient variables significantly increased (Fig. 9; Table 2). When PMCA activity was restored by returning bath pH to 7.3, resting fluorescence and all four transient variables returned to baseline levels.

**Table 2.** Decreased PMCA activity increases GECI calcium transients

	pH 7.3	pH 8.8	pH 7.3
Resting F (a.u.)	583 ± 86 (22)	661 ± 143 (22)***	580 ± 80 (22)
Peak (%ΔF/F)	28 [18–71] (22)	55 [25–84] (22)**	23 [14–49] (22)
RT (s)	1.21 ± 0.27 (21)	1.63 ± 0.36 (21)****	1.17 ± 0.28 (21)
DT 1 (s)	2.77 ± 0.82 (19)	4.71**** ± 1.78 (19)****	2.22 ± 0.47 (19)
DT 2 (s)	8.78 ± 0.99 (16)	10.49** ± 2.30 (16)**	7.88 ± 1.56 (16)

Median [IQR] or mean ± SD. Parentheses indicate number of cells. Different from baseline: **p < 0.01, ***p < 0.001, ****p < 0.0001. Changes in calcium transient parameters in response to elevation of bath pH to block PMCA activity. Paired *t* tests revealed that parameter values significantly increase when bath pH is changed to pH 8.8 and recover when bath pH is returned to pH 7.3.

## Discussion

### Investigating PV+ DRG neuron calcium homeostasis with a GECI

This study investigated the calcium dynamics of PV+ DRG neurons using the GECI, GCaMP6s, in transgenic mice. Selective expression of GCaMP enabled the identification of proprioceptive neurons from other DRG neuron populations. Profiling calcium dynamics with optical recordings can facilitate greater yield in terms of cells analyzed per preparation than intracellular recordings.

While cell-type specific expression is a major benefit of GECIs, inherent differences between and within subsets of neurons may influence reporter efficacy. Dozens of PV+ neurons in the DRG responded to nerve stimulation in each preparation, but it is likely that some electrically active PV+ neurons were undetectable with calcium imaging. Studies using synthetic calcium indicators have shown that large-diameter DRG neurons, which would include PV+ neurons, have smaller amplitude and more rapidly decaying transients than small-diameter DRG neurons (Lu et al., 2006; Fuchs et al., 2007). In fact, among Aβ neurons, low-threshold mechanoreceptors that will include some PV+ neurons, calcium transients from single action potentials were undetectable in 50% of the cells studied (Gemes, et al., 2010). Both Aβ and proprioceptive afferents have much shorter action potential durations than small-diameter DRG neurons (Koerber and Mendell, 1992), thus limiting the time window for calcium entry during an action potential. Strong intrinsic calcium buffering capacity provided by PV expression also likely diminishes the fraction of responding cells in these preparations. The presence of GCaMP6s in PV+ neurons will also increase the buffering capacity for intracellular free calcium in these neurons. While buffering by GCaMP6s is an important experimental variable to consider in our analysis, this caveat applies to any calcium indicator, GECI or synthetic, that requires binding of calcium to generate fluorescence (McMahon and Jackson, 2018). Calcium indicators in general alter the kinetics of cellular calcium fluxes, but unlike short-term *in vitro* experiments with synthetic indicators, long-term expression of GCaMP6s in our system may influence expression or function of endogenous buffering mechanisms. Nevertheless, our results demonstrate that use of a GCaMP6s in PV+ neurons has sufficient resolving power to detect pharmacologically induced alterations.

### PV+ DRG neuron calcium transient properties

Our data support the hypothesis that GECI calcium transients can be used in a similar manner to synthetic indicators for studying calcium dynamics of PV+ neurons. Linear regressions for peak amplitude, RT, DT1, and DT2 all produced small *R* ^2^ values, demonstrating that each parameter can only predict a small portion of variability for the other parameters. For example, peak amplitude was predictive for only 8% of DT1 variability (*p* < 0.0001, *R* ^2^ = 0.08), demonstrating that these two characteristics vary independently from each other in most cells. This finding is consistent with studies using synthetic indicators, which demonstrated that decay time is not affected by peak amplitude (Duncan et al., 2013).

The largest *R* ^2^ value was 0.28 between DT1 and RT (*p* < 0.0001). DT1 is largely a function of the sequestration and extrusion of calcium via SERCA and PMCA pumps. These two calcium-regulating mechanisms were shown to be responsible for the majority of the decay time in studies that used synthetic dyes on DRG neurons (Benham et al., 1992; Usachev et al., 2002; Lu et al., 2006), although mitochondrial uptake and extrusion through plasma membrane sodium-calcium exchangers also contribute to recovery times with large calcium loads (Lu et al., 2006). Indeed, when SERCA was blocked with TG in this study, DT1 increased 111% from baseline. RT also increased with TG, but only by 59%. These findings demonstrate that both DT1 and RT are influenced by SERCA, but not to the same degree. Additional mechanisms, such as calcium-induced calcium release, may affect RT, but not the time course of extrusion mechanisms. Taken together with the linear regression results, it is likely that multiple calcium-regulatory mechanisms contribute to each of these variables, with some overlap.

A linear regression between DT1 and DT2 demonstrated that the two recovery variables could not predict each other (*R* ^2^ = 0.04, *p* = 0.006). Thus, it is likely that this second, residual part of the transient recovery is regulated by additional or different calcium regulatory machinery than DT1.

The average RT of calcium transients in our experiments was 1.2 s, which exceeded the duration of the standardized 0.5-s stimulus. The slow kinetics intrinsic to GECIs, combined with the properties of the slow (s) variant of GCaMP6, likely summate and together contribute to the extended RTs found in this study. Previous reports described GCaMP6s RTs of ∼0.5 s after stimulation with a single action potential compared to <0.2 s with GCaMP6f (fast variant) and <0.05 s with OGB1, a synthetic calcium indicator (Chen et al., 2013).

The decay of calcium transients in this study was best fit by a second-order exponential decay function, yielding an average decay constant of 2.6 s (DT1; time required to decay 63% from the peak). While other studies using GCaMP6s have not specifically reported decay constant values, GCaMP6s transients in cultured hippocampal neurons decayed to 50% of peak amplitudes in ∼2.5 s when subjected to stimuli protocols similar to ours (25 action potentials at 83 Hz; Chen et al., 2013). When these experiments were repeated with OGB1, decay times were slightly <1.0 s (Chen et al., 2013). Thus, the DT1 values found in this study align with those found in the literature and are influenced by kinetic properties of the GCaMP6s indicator.

The peak amplitude of calcium transients demonstrated more variability than other transient parameters and the pooled data were skewed toward larger values, unlike the relatively normal distributions for resting fluorescence, RT, DT1, and DT2. Another study that used GCaMP6s in mouse DRG neurons also reported wide variability in peak amplitudes between cells, with average peak amplitudes of 654 ± 974% (mean ± SD; Chisholm et al., 2018). One possible explanation for such variability in peak amplitude could be intrinsic differences in GCaMP6s expression between cells. However, resting fluorescence, an indirect measure of fluorescent indicator expression levels, did not exhibit the same degree of variability as peak amplitude and the majority of resting values could not be predicted by peak amplitude. Furthermore, one study examined the effects of varying expression levels of GCaMP and found no differences in peak amplitude or other transient characteristics across a 3-fold range in expression (Lock et al., 2015). While the GCaMP expression levels of individual cells could not be measured in this study, given our resting fluorescence values and the results from Lock et al. (2015), it is unlikely that differences in GCaMP expression levels produced the variability in peak amplitudes found in this study.

Another explanation is that the variability in peak amplitudes between cells and animals reflect genuine differences in cytosolic calcium fluctuations, as peak amplitudes changed in response to manipulations of electrical activity and extracellular calcium concentration. Furthermore, a given cell could produce a range of peak amplitudes, from 4% to 400%ΔF/F depending on electrical stimuli and bath calcium concentration. This demonstrates that neither limitations in GCaMP expression levels nor imaging factors restrict the fluorescence range of a cell. Also, in experiments where cells were repeatedly imaged over several hours, peak amplitudes for a given cell at a given stimulus changed on average <15% over time. Taken together, the cell-to-cell variability in peak amplitudes reported here is most likely due to biological variation in the intrinsic physiologic properties of PV+ neurons that influence the magnitude of calcium fluctuations during electrical activity. Future studies that image cells before and after the addition of drugs, injuries, or other manipulations can interpret changes of <15% from baseline transients to represent normal variation.

### GECI calcium transients change with alterations to electrical activity and external calcium concentrations

GECIs will respond in a non-linear fashion to calcium fluctuations if the changes are relatively large or small (Rose et al., 2014). However, in theory, changes in GCaMP fluorescence should directly correspond with fluctuations in intracellular calcium when calcium fluctuations are intermediate in size.

In normal calcium ACSF (2 mM), trains of 5–100 action potentials at 50 Hz produced incremental increases in peak amplitude, consistent with a linear relationship between GCaMP fluorescence and action potential number. Manipulating extracellular calcium concentrations reaffirmed these findings. In high-calcium conditions (4 mM), peak amplitudes of transients were slightly higher than in normal calcium (2 mM) at each measured condition, i.e., 1–100 action potentials at 50 Hz. Low-calcium conditions (no calcium added) generated peak amplitudes that were smaller than both normal and high-calcium conditions for every stimulation protocol. The fact that GECI transients could detect these changes in calcium fluctuations demonstrates that, for PV+ DRG neurons, our stimulation protocol of 25 action potentials at 50 Hz is sufficient to reliably detect changes in intracellular calcium fluctuations. Furthermore, these findings present evidence that with a stimulation of 25 action potentials at 50 Hz, the calcium fluctuations fall within the linear range of GCaMP6s, reinforcing the conclusion that the variability in peak amplitudes across cells and animals reflect genuine differences in calcium dynamics.

GECI peak amplitudes also grew with increasing stimulation frequency up to 150 Hz, and then decreased or plateaued with higher frequency stimulation. At the lowest frequency examined, 1 Hz, we could not reliably identify individual spikes in the calcium transient (individual spikes observed in one of four cells examined). Discernable individual transients at low frequencies (0.5–2 Hz) have been reported using confocal microscopy and GCaMP6s in DRG neurons, but individual spikes were less apparent with two-photon microscopy (Chisholm et al., 2018). It should be noted that the firing rate of proprioceptors, the majority of PV+ DRG neurons, averages more than 50 Hz during stretch (Matthews, 1972). While spike inference was not critical in this study, answering research questions related to stimulus encoding will be limited until GECIs or post-processing algorithms can provide sufficient temporal resolution to decipher physiologically relevant frequencies for PV+ neurons.

Our findings that GECI peak amplitudes increase with rising firing frequency, electrical activity, and intracellular and extracellular calcium concentrations are corroborated in a recent study using a Drosophila model and the GCaMP6m variant, as well as GCaMP1.3 and myrGCaMP5 (Xing and Wu, 2018). Thus, growing evidence suggests that GECIs can provide meaningful insights into the magnitude of calcium fluctuations despite their non-linear response curves in certain conditions.

### Assessing calcium pump function with GECIs

Blocking SERCA activity with TG significantly increased the RT and DT1 of calcium transients, but had no effect on resting fluorescence, peak amplitude, or DT2. These results suggest that in PV+ neurons, both the rise phase and initial decay phase of GECI calcium transients are influenced by the activity of SERCA. Studies using synthetic indicators also found that blocking SERCA increased decay time without affecting peak amplitude (Sasaki et al., 2008; Gemes et al., 2012).

In a second set of experiments, bath pH was increased from 7.3 to 8.8 to inhibit PMCA activity. In contrast to blocking SERCA, blocking PMCA significantly increased all calcium transient parameters demonstrating that PMCA activity affects every aspect of GECI transients in PV+ neurons. Studies using synthetic dyes also found that inhibiting PMCA activity by elevating bath pH increased peak amplitude and decay times (Gemes et al., 2012; Duncan et al., 2013). In addition to corroborating previous studies, our finding that resting fluorescence significantly increased with decreased PMCA activity indicates that GECIs are sensitive enough to detect changes in resting calcium concentrations. In small DRG neurons *in vitro*, resting calcium rose from ∼100 to 150 nM with TG and with increased bath pH (Duncan et al., 2013). In the present study, resting fluorescence only significantly changed with elevated bath pH, and not with TG. It is possible that resting calcium concentration is not affected by TG in the subpopulation of PV+ neurons, or not affected at levels detectable by GECIs, which underestimate very small changes in calcium. However, the ability to detect changes in resting fluorescence was consistent when PMCA was manipulated. A total of 20 of 22 cells exhibited increased resting fluorescence when PMCA was inhibited, and resting fluorescence decreased back to original values when PMCA activity was restored by returning pH to 7.3. Thus, GECIs consistently report changes in resting calcium levels when the changes are sufficiently high for detection by GECIs.

Taken together, these experiments provide strong evidence that the relationships between SERCA or PMCA activity and GECI calcium transient parameters are causal. Furthermore, the measured changes in GECI transient parameters in response to manipulations of calcium regulatory machinery were robust and pervasive across cells; changes from baseline recordings were statistically significant with a small number of cells (<20). Thus, GECI calcium transients provide sufficient resolution to discern information about the activity of specific calcium regulatory mechanisms.

By comparing and contrasting the results from inhibiting two different calcium-regulating mechanisms, we provide insights into these mechanisms in PV+ neurons for the first time. For example, because PMCA activity affects all phases of the calcium transients in this study, we can infer that PMCA is active both at rest and during stimulation in PV+ neurons, as in other DRG populations (Gemes et al., 2012). Conversely, SERCA activity only affected RT and DT1 in GECI transients. Before this, studies have not reported that SERCA activity affects the RT of calcium transients. Given the slow kinetics of GCaMP6s, it is likely that SERCA begins sequestering calcium while the GECI transient is still rising, whether or not cytosolic calcium concentrations are still increasing. Therefore, when SERCA sequestration is blocked, more calcium would remain in the cytoplasm, prolonging the RT and DT1 of the GECI transient. That blocking PMCA also prolongs RT supports the inference that blocking calcium removal pathways can lengthen RT. Interestingly, peak amplitude increased with PMCA inhibition but not with SERCA inhibition. Linear regressions demonstrated that peak amplitude varied independently from RT in most cells and therefore is likely controlled by different calcium regulatory mechanisms. Thus, when SERCA is blocked, another calcium regulatory mechanism, possibly PMCA, may be maintaining peak amplitude. The critical finding from these experiments is that blocking two different calcium regulatory mechanisms generated two distinct sets of changes to GECI transients, demonstrating that GECIs can provide insights into the activity of specific calcium regulatory machinery.

## Synthesis

Reviewing Editor: Lorna Role, NINDS Decisions are customarily a result of the Reviewing Editor and the peer reviewers coming together and discussing their recommendations until a consensus is reached. When revisions are invited, a fact-based synthesis statement explaining their decision and outlining what is needed to prepare a revision will be listed below. The following reviewer(s) agreed to reveal their identity: Sarah Canetta, Michael Gold.

Overall the reviewers felt that the revisions were satisfactory in that they have addressed previous concerns regarding the utility of using GECIs to assess calcium activity in sub-populations of DRG neurons expressing PV. Although the authors do not directly demonstrate that they can use this technique to study how different perturbations/alterations may affect PV+ neuronal calcium regulation, this article is now stronger as a foundational paper for establishing the technique itself.

One issue that must be addressed is for the manuscript to be brought up to date with statistical reporting practices. In this regard, the details regarding the statistical tests used throughout is still minimal at best. The authors should clarify for each p-value provided the statistical test used and in the case of ANOVA's provide the results of the ANOVA as well as the posthoc tests employed.

Additional Minor comments:

On page 12, line 282, the statistics suggest an R2 of 0.088 between peak amplitude and resting fluorescence but the R2 listed in the figure (2H) is 0.10.

For Figure 6, There seems to be some confusion with respect to the stimulation paradigm used. The authors' reference in yellow to the ‘standard stimulation of 25 APs used elsewhere in this study’ (also highlighted in Figure 5 in yellow). But according to the methods and the figure legend, in Figure 6, the yellow stimulation would be 25 Hz stimulation delivered for 0.5s, which would be expected to result in 12 APs, rather than 25, unless each stimulus generates 2 APs? Please clarify.
